# Patient’s and healthcare provider’s experiences with Opioid Maintenance Treatment (OMT): a qualitative evidence synthesis

**DOI:** 10.1186/s12913-024-10778-7

**Published:** 2024-03-13

**Authors:** Asbjørn Steiro, Christine Hillestad Hestevik, Ashley Elizabeth Muller

**Affiliations:** Department of Health Services Research, Norwegian Institute of Public Health, Pb 222, 0213 Skoyen, Oslo, Norway

**Keywords:** Opioid maintenance treatment, User experience, Patient and health providers, Qualitative evidence synthesis, Opioid-related disorders, Health policy

## Abstract

**Background:**

Opioid Maintenance Treatment (OMT) is the gold standard for people with opioid dependence. However, drop-out rates are high, and many patients do not reach desired outcomes. Understanding patients’ and healthcare providers’ experiences with the treatment can provide valuable information to improve the quality of OMT and to increase acceptability and accessibility of services. The aim of this systematic review is to explore and synthesise the experiences of OMT among persons with opioid dependence and health care providers, to inform policy makers and practitioners on how to improve OMT outcomes.

**Methods:**

We conducted a qualitative evidence synthesis. We systematically searched in electronic databases (CINAHL, Embase, MEDLINE, and nordic databases) and searched for grey literature. As we identified many studies that met our inclusion criteria, we purposively sampled a manageable number of studies to include in this review. Two researchers independently extracted and coded data from the included studies and used the Andersen’s healthcare utilization model to organize and develop codes. We assessed the methodological limitations of the studies, and our confidence in the findings using GRADE CERQual.

**Results:**

We retrieved 56 relevant studies and purposively sampled 24 qualitative studies of patients’ and healthcare providers’ experiences with OMT. Our analyses resulted in six main themes: (1) External stigma prevents engagement and retention in treatment, (2) Being identified as in OMT contributed to an increased experience of stigma (3) Inadequate knowledge and expertise among healthcare providers affected patients’ treatment experiences, (4) Quality of communication between personnel and patients impacts patients’ engagement with treatment and treatment outcomes, (5) Patients wanted help with many aspects of their lives not just medication, and (6) Balancing positive expectations of OMT with treatment stigma. We found that stigma was an overarching theme across these themes.

**Conclusion:**

Our findings suggest that OMT could be more beneficial for patients if treatment programs prioritize efforts to diminish societal and OMT provider stigma and find strategies to better address patient needs. Initiatives should focus on improving treatment knowledge among providers, encouraging the use of client perspectives, considering the context of family members, and establishing a more holistic and flexible treatment environment.

**Supplementary Information:**

The online version contains supplementary material available at 10.1186/s12913-024-10778-7.

## Background

People with opioid dependence are nearly 15 times more likely to die prematurely than people without opioid dependence [1]. Causes of death vary and commonly include suicide and traumatic incidents, with drug overdose being the most prevalent cause of death. Deaths among people with opioid use disorders results in several decades of lost life [2].

Opioid maintenance treatment (OMT) is the first choice of treatment for persons with opioid dependence in many countries [3–8]. OMT is a comprehensive rehabilitation course where opioid agonists, antagonists, and partial agonists (mainly methadone or buprenorphine) are used to substitute the use of harmful non-medical use of illegal opioids, such as heroin, with the objective of stabilizing the patient and alleviating symptoms of abstinence or intoxication [7, 9–11]. Other important goals of the treatment OMT are to improve health and social functioning (such as housing, education, employment, integration in drug-free networks and contact with family. The medicinal aspect of treatment is typically complemented by psychosocial support and rehabilitation services, to address both the physical and social aspects of opioid use disorder [12]. However, globally the contents of OMT programmes vary. They differ in use of primarily medications as well as, and resources used on for psychosocial rehabilitation and treatment of comorbid disorders [12].

The beneficial effects of OMT compared to not being in OMT are well documented: patients remain in treatment longer, use less heroin and other non-prescribed opioids, and all types of mortality are reduced [13–15]. A longitudinal study that included all persons in Norway applying for and entering OMT between 1997 and 2009 (*n* = 6871) found that being on OMT significantly reduced the risk of mortality compared to being outside of treatment throughout the whole observation period [16].

However, many patients do not reach desired outcomes in OMT. Drop-out rates are high: about one-third of patients in Norway leave within the first 18 months, despite OMT being intended to be a long-term treatment [17]. Dropping out of OMT is associated with heightened risks of mortality and overdose [18, 19]. A recent systematic review that assessed factors associated with retention in OMT, found that younger age, substance use particularly cocaine and heroin, lower doses of methadone (a wide variability in measurement of dosage was reported), criminal activity or incarceration, and negative attitudes towards the treatment seemed to be associated with reduced retention in treatment [20]. They also found that retention rates decreased with time in OMT and fell from approximately from 57% at 12 months to 38.4% at three years [20].

Previous qualitative findings also emphasize on the difficulty of the treatment experience for patients in OMT. For example, the study by Neale and colleagues [21] described how some patients feel “trapped” in treatment, and that OMT causes another form dependency that leaves them in a stigmatized position. Others have used the metaphor of “liquid handcuffs” [22].

Ahern and colleagues interviewed 1008 American illicit drug users about stigma and discrimination related to their drug use. They found that the stigma appeared to increase when patients accepted treatment and were labelled with a formal diagnosis [23]. They also found that stigma and discrimination was associated with poorer mental health. In a mixed method systematic review, researchers found that stigma from family, friends, and healthcare providers posed significant barriers to accessing OMT. Stigma was also intertwined with concerns about job opportunities and the fear of being identified at the treatment center [24].

In 2019, the Norwegian Directorate of Health planned to update the national clinical practices guideline for OMT and commissioned a qualitative evidence synthesis (QES) of patients’ and healthcare providers’ experiences with OMT to inform the guideline updates [25].

The commission of this QES was important, as it highlighted the centrality of patients’ and healthcare providers’ experiences within this treatment, and not only outcomes such as substance use and adherence. This QES has only been published in Norwegian, and we believe that this topic would be of interest to the global society of researchers and clinicians and policymakers within the field of OMT. Therefore, we have updated the original QES in English.

Synthesized research with a cross study approach that assess confidence in the qualitative data can provide valuable information on how to improve OMT and treatment outcomes [26]. Numerous quantitative systematic reviews had been conducted on OMT, however, there seem to be few qualitative reviews that explore the experiences of OMT patients or healthcare providers. This QES seeks to fill this knowledge gap in the field. The experiences of patients in OMT and their healthcare providers have a significant importance that can generate knowledge on how to improve acceptability, access to and usage of OMT services.

## Methods

This QES followed the best practice as described by the Cochrane collaboration in their handbook [27, 28]. We systematically searched in electronic databases, identified studies that met our inclusion criteria, extracted, and coded data from the included studies, assessed the methodological limitations of the included studies, and our confidence in the findings using the GRADE CERQual approach. We have also used the Enhancing transparency in reporting the synthesis of qualitative research: ENTREQ checklist (appendix 1).

### Study identification

A research librarian planned and conducted systematic searches in seven electronic databases (CINAHL (EBSCO), Embase (Ovid) Ovid MEDLINE, and Nordic databases (SveMed+, Norart, Idunn, Oria, Nora, Oda) in September 2019. The same librarian updated this search in November 2021. As evidence in this field also consists of studies that are not published in commercial publications, such as masters- and PhD thesis, we also conducted a search for grey literature in Google Scholar in January 2022 using the search terms opioid maintenance and *qualitative study*, restricted to studies published from 2019 to 2022. In addition, we examined reference lists of relevant reviews.

Two researchers independently screened titles and abstracts, then relevant full-text studies, for adherence to the following inclusion criteria: Qualitative studies portraying views, perceptions, and expectations of OMT among adult patients with opioid dependency and healthcare providers in OMT. We used the systematic review management program Covidence 4 in the selection process [29].

### Sampling studies

We initially identified 56 studies that met our inclusion criteria. We extracted the following data from these studies: First author, year, population, context, research questions, main findings. One author extracted data and another author checked the data extraction.

After we mapped the 56 studies, we realized that the amount of data from the studies would make analysis and synthesis unmanageable. The quality of synthesis may be compromised when dealing with a substantial number of primary qualitative studies and a high volume of data [26, 30]. The analysis and synthesis of qualitative data demand a meticulous engagement with the text and challenges arises when confronted with large volumes of data, as less depth of understanding is likely to be gained from the data [26, 31, 32]. To ensure that we included a manageable number of studies aligned with our research objectives, while also ensuring geographical representation and rich data, we conducted a purposive sampling of studies used a sampling frame based on information obtained during data extraction. Following the methodology developed by Ames and colleagues [26], we developed a sampling frame based on the needs of our review question. First, we assessed the relevance of the studies to our research question and assessed their data richness using a data richness scale [26]. Second, we selected studies that represented variations in geographical areas (countries), settings (general practitioner office, methadone centres, pharmacies, OMT medication) and study populations (gender, age). Finally, we also evaluated the transferability of the studies to the Norwegian setting, leading us to only include studies conducted in middle to high-income countries.

**Fig. 1:**
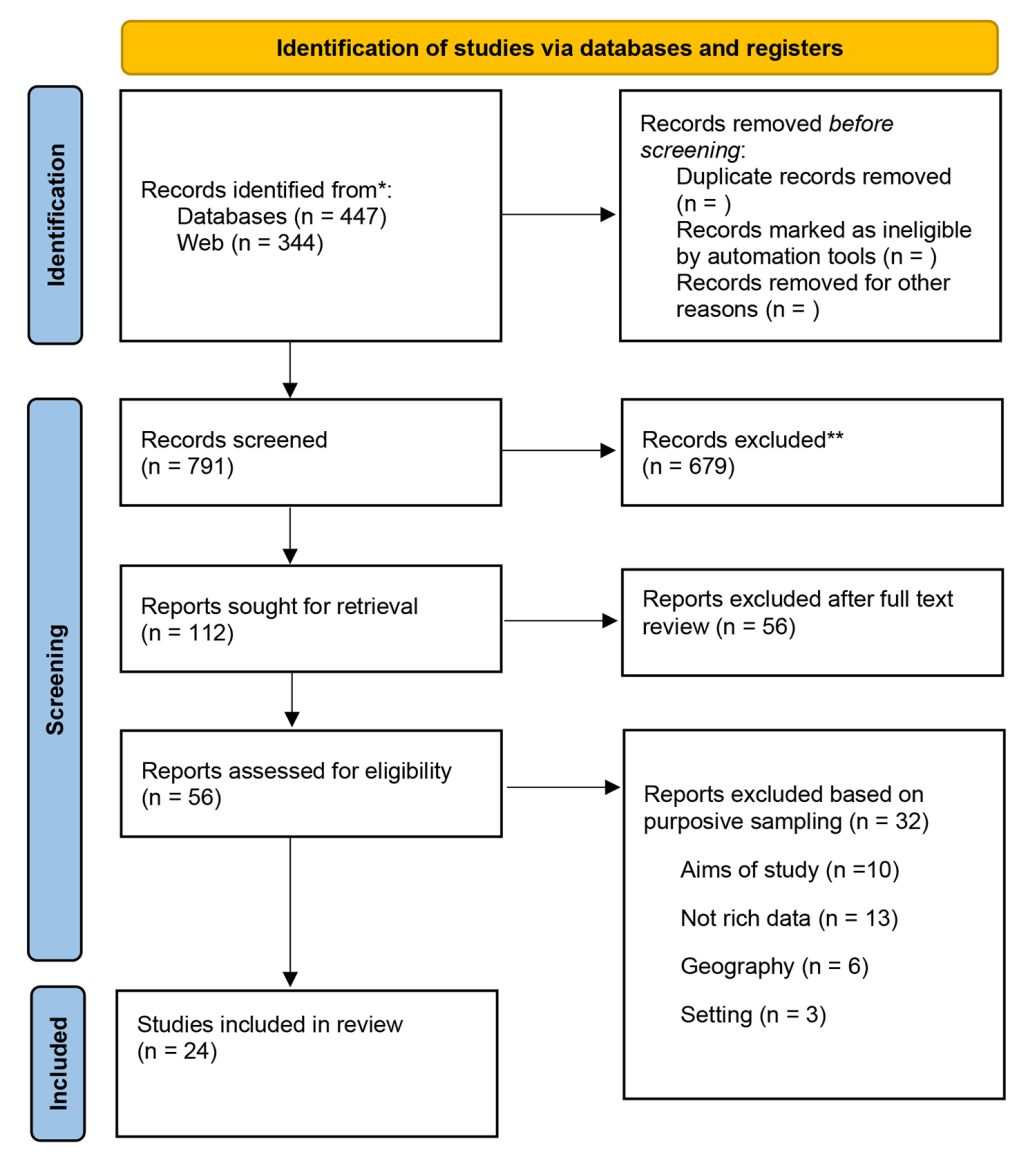
**PRISMA flow diagram for selection of studies** Legend: Retrieved From: Page MJ, McKenzie JE, Bossuyt PM, Boutron I, Hoffmann TC, Mulrow CD, et al. The PRISMA 2020 statement: an updated guideline for reporting systematic reviews. BMJ 2021;372:n71. doi: 10.1136/bmj.n71. For more information, visit: http://www.prisma-statement.org/ *Consider, if feasible to do so, reporting the number of records identified from each database or register searched (rather than the total number across all databases/registers) **If automation tools were used, indicate how many records were excluded by a human and how many were excluded by automation tools *From*: Page MJ, McKenzie JE, Bossuyt PM, Boutron I, Hoffmann TC, Mulrow CD, et al. The PRISMA 2020 statement: an updated guideline for reporting systematic reviews. BMJ 2021;372:n71. doi: 10.1136/bmj.n71 For more information, visit: http://www.prisma-statement.org/

Three researchers independently applied the sampling frame. We then compared our included studies. When discrepancies arose, these were solved through discussion to reach a consensus. Twenty four of the 56 studies that met our inclusion criteria were included in the synthesis (Fig. 1).

### Critical appraisal

We assessed the methodological limitations of the included studies using the Critical Appraisal Skills Programme (CASP) [33]. CASP contains ten questions to assess the following domains: (1) clear statement of the aim of the studies, (2) was the qualitative methodology appropriate, (3) research design, (4) recruitment strategy, (5) data collection, (6) consideration of relationship between researchers and participants (7) ethical issues, (8) rigour data analysis, (9) clear statement of findings, and (10) value of the research. We appraised the quality of the documentation from each included study as low, moderate or high according to these criteria.

Each study was individually appraised by two authors (AS and CHH). Disagreements among the authors were solved by discussion or by conferring with a third author (AEM).

### Qualitative synthesis

We used a two-step analytic procedure. First, two researchers individually coded the data inductively, before coordinating the coding and agreeing on emerging topics across the included studies. Second, two other researchers independently checked and refined these topics and examined whether a framework could be helpful in illustrating our results. We found that the Andersen’s healthcare utilization model [34] would be useful to illustrate and explain the dynamic of factors that facilitate or inhibit provision of OMT and patients’ access to and usage of OMT. We used this model to organize and develop the inductively created codes into four main domains: contextual factors, individual factors, health behavior and outcomes/results of treatment. The aim of Andersen’s model is to identify factors that lead to the use of health services. According to the model, usage of health services in these domains can be understood as influenced by three dynamics:

1. Predisposing factors: Individual predisposing factors (demographic characteristics of age and sex as “biological imperatives”), social factors (education, occupation, ethnicity and social relationships), mental factors in terms of health beliefs (e.g., attitudes, values, and knowledge related to health and health services) and contextual factors (demographic and social composition of communities, collective and organizational values, cultural norms, and political perspectives).
2. Enabling factors: Financing and organizational factors that serve as conditions for enabling services utilization.
3. Need factor: perceived need for health services (i.e., how people view and experience their own general health, functional state and illness symptoms) and evaluated need (i.e., professional assessments and objective measurements of patients’ health status and need for medical care). A persons need for care motivates service use. For example, psychological and physical conditions, illness or disease conditions.

We mapped our codes onto this framework, and then re-examined these codes to pay attention to themes that did not fit, or that could be cut cross all the four domains on Andersen’s model. Stigma was an example of a topic that cut across all four domains. A classic best fit analysis would have suggested for stigma to be added to this four-domain framework, but our approach was considered more suitable for our analysis. We used the NVivo program for data management [35].

### Confidence in the findings

We used the Confidence in the Evidence from Reviews of Qualitative research (GRADE-CERQual) approach [36–42]. The GRADE-CERQual approach is a way to determine how confident we can be in QES findings. Four components are assessed: Methodological limitations of included studies, coherence of the review finding, adequacy of the data contributing to a review finding, and relevance of the included studies to the review question. Each finding is classified as having “no concerns”, meaning we are confident that this finding can be used in decision-making, to “serious concerns”, indicating the opposite. We also assessed our overall confidence in the evidence supporting each review finding, as high, moderate, low, or very low (Table 1). Individual and comparative appraisals were discussed among the authors until an agreement was reached.

**Table 1:** Descriptions of level of confidence in a review finding in the GRADE-CERQual approach (Lewin et al., 2015)

Level	Definition
High confidence	It is highly likely that the review finding is a reasonable representation of the phenomenon of interest
Moderate confidence	It is likely that the review finding is a reasonable representation of the phenomenon of interest
Low confidence	It is possible that the review finding is a reasonable representation of the phenomenon of interest
Very low confidence	It is not clear whether the review finding is a reasonable representation of the phenomenon of interest

## Results

We read the full text of 56 studies and carried out a purposive sampling of 24 studies which we included in this review (Fig. 1). There were 13 studies in which the participants were patients in OMT [43–55], 8 in which the participants were healthcare providers [56–63], and 3 studies included the experiences of both patients and healthcare providers [64–66]. The studies were conducted in the USA [6], United Kingdom [5], Canada [5], Norway [3], Sweden [2], Belgium [1] and New Zealand [1]. See appendix 2 for description of the included studies.

Our analysis resulted in six main themes:

(1) External stigma prevents engagement and retention in treatment. (2) Being identified as in OMT contributed to an increased experience of stigma. (3) Inadequate knowledge and expertise among healthcare providers affected patients’ treatment experiences (4) Quality of communication between personnel and patients impacts patients’ engagement with treatment and treatment outcomes. (5) Patients wanted help with many aspects of their lives not just medication. (6) Balancing positive expectations of OMT with treatment stigma.

We employed Andersen’s model as a frameworkto understand how contextual and individual factors, health behaviours, and outcomes serve as facilitators or barriers to predispose and enable service utilization. Notably, stigma emerged as a pervasive theme cutting across all these domains. Particularly, the stigma originating from individuals outside the OMT community acted as a significant barrier, impeding both the initiation and sustained engagement in treatment.

The themes and our confidence in each finding are presented in Table 2.

**Table 2:** CERQual Qualitative Evidence Profile– overall assessment of confidence

Finding	CERQual- assessment*	Explanation of assessment	Contributing studies
1. External stigma prevents engagement and retention in treatment	High confidence	Methodological limitations: No or minor concerns. Coherence, relevance and the fit of the data: Minor concerns.	15 studies Bates 2021; Bishop 2019; De Maeyer 2011; Gordon 2011; Granerud 2015; Green 2014; Harris 2015; Hewell 2017; Korthuis 2010; Livingston 2018; Richert 2015; Silva 2021, Tanner 2011; Toft 2013
2. Being identified as in OMT contributed to an increased experience of stigma	High confidence	Methodological limitations: No or minor concerns. Coherence, relevance and the fit of the data: Minor concerns.	17 studies Bates 2021; Belseth 2016; Bishop 2019; De Maeyer 2011; Gordon 2011; Granerud 2015; Green 2014; Harris 2015; Hewell 2017; Korthuis 2010; Marchand 2020; Notley 2014; Notley 2015;; Silva 2021; Tanner 2011; Woo 2017; Yadav 2019
3. Inadequate knowledge and expertise among healthcare providers affected patient’s treatment experience	Moderate confidence	Methodological limitations: Minor concerns.Coherence and relevance: No or minor concerns. The fit of the data: Moderate concerns.	12 studies Bates 2021; Gordon 2011; Granerud 2015; Green 2014; Hewell 2017; Johnson 2014; Lachapelle 2021; Livingston 2018; Notley 2014; Van Hout 2018; Woo 2017, Yadav 2019
4. Quality of communication between personnel and patients impacts patients’ engagement with treatment and treatment outcomes	High confidence	Methodological limitations: Minor concerns.Coherence , relevance and the fit of the data: No or minor concerns.	18 studies Belseth 2016; Bishop 2019; De Maeyer 2011; Gordon 2011; Granerud 2015; Green 2014; Harris 2015; Hewell 2017; Korthuis 2010; Lachapelle 2021; Livingston 2018; Marchand 2020; Notley 2014; Notley 2015; Silva 2021; Tanner 2011; Toft 2013; Yadav 2019
5. Patients wanted help with many aspects of their lives not just medication	Moderate confidence	Methodological limitations: Moderate concerns. Coherence and the fit of the data: No or minor concerns. Relevance: Moderate concerns.	15 studies Bishop 2019; De Maeyer 2011; Granerud 2015; Harris 2015; Hewell 2017; Korthuis 2010; Lachapelle 2021; Notley 2014; Notley 2015; Richert 2015; Silva 2021; Sohler 2013; Tanner 2011; Van Hout 2018; Yadav 2019
6. Balancing positive expectations of OMT with treatment stigma .	Low confidence	Methodological limitations: Moderate concerns. Coherence and the fit of the data and relevance: serious concerns	8 studies Bates 2021; De Maeyer 2011; Harris 2015; Granerud 2015; Green 2014; Notley 2015; Silva 2021; Tanner 2011

* High confidence: it is highly likely that the review finding is a reasonable

Medium confidence: it is likely that the review finding is a reasonable

Low confidence: it is possible that the review finding is a reasonable

Very low confidence: it is not clear whether the review finding is a reasonable

See appendix for supplementary material

### Finding 1: External and internal stigma prevents engagement and retention in treatment

Stigma appeared immediately as a *contextual* barrier, attitudes towards OMT in the social context surrounding the individual. Stigma among families, friends, and in society in general was a strong negative influence on the persons’ decision to start OMT [46, 50, 51, 54]. While many participants considered being in OMT as a better position than being outside OMT [43–45, 47, 54, 64], stigma reported by other participants meant that this was not always the case. Stigma could also serve as a link between health perceptions in society and the individual’s own health perceptions [53].

This contextual barrier became an *individual* factor in the form of internalized stigma. Some patients had internalized this external stigma in the form of shame [44, 46, 50, 51]. Beginning in OMT was a public declaration of one’s opioid use disorder. In some studies, people escaped or avoided the stigma of making this public declaration by not seeking treatment and keeping opioid use hidden. When they began in OMT, they accepted the stigma conferred by it [46, 50].

Stigma could also be a barrier for healthcare providers to provide OMT. One clear attitude was that healthcare providers viewed persons in OMT as difficult patients. Persons in OMT were expected to be demanding and to “scare away” other patients [56, 58, 61, 64]. Another stigmatizing attitude was that some healthcare providers outside the OMT system believed that OMT was not a treatment, but just a different type of addiction [60]. They were negatively tuned to OMT and believed it was the wrong approach to treat opioid addiction. This attitude towards OMT led them to actively discourage or disrespect OMT as a treatment option [56, 59].

### Finding 2:Being identified as in OMT contributed to an increased experience of stigma

Our second finding relates to stigma as an experience of OMT itself. This should also be seen in the context of external factors. It was not only families, friends, and the community at large that saw OMT as something negative [43, 44, 46, 55]. Employees at pharmacies, healthcare providers, and others who were part of the OMT system also stigmatized patients. Their beliefs were rooted in the societal perceptions of persons with opioid disorders and those in OMT [56]. On the other hand, healthcare providers themselves influenced society’s perceptions [58, 59, 63, 64]. When healthcare providers reflected these negative attitudes from ‘outside’ the treatment system, patients expected the OMT program to stigmatize them further [45, 55].The results suggest that OMT contributed to further stigma when recipients of treatment, according to both patients and healthcare providers, were treated and labelled by their condition and not as “ordinary” patients [45, 51]. Additionally, perceived acceptance and warmth displayed by healthcare providers were considered to be of great importance for treatment success [49, 51].

In light of Andersen’s model, stigma from the OMT system had a distinctly negative effect on how willing patients were to remain in OMT. In the end, the stigma was reproduced by the OMT system, which is an outcome beyond relationships between the patient and healthcare providers. The way OMT was physically/spatially organized in some cases meant that patients felt more stigmatized, sometimes to an even greater extent than when they were outside OMT [46, 47, 64, 66]. For example, some patients experienced having to stand in line with other OMT patients to pick up their medications at OMT specific pick-up points, as more stigmatizing than picking up a prescription at the pharmacy along with other customers [47, 64]. Many patients also rated patients receiving methadone lower than those receiving buprenorphine. They regarded patients on methadone as “real abusers” [43, 44, 53, 55, 64].

### Finding 3 Inadequate knowledge and expertise among healthcare providers affected the patients’ treatment experience

We identified a lack of knowledge and expertise among healthcare providers as contextual factors that influenced the quality and availability of OMT. Patients and healthcare providers reported that inadequate knowledge and expertise among healthcare providers were barriers, which affected access to OMT, the quality of treatment, patients’ experiences, and treatment outcomes [45, 46, 55, 56, 60, 63]. They also reported that inadequate knowledge and expertise regarding the client population and treatment affected their ability to provide quality care [45, 46, 55, 56, 60, 63]. This may also have contributed to negative attitudes towards OMT patients and OMT among healthcare providers.

The need for adequate knowledge, access to experts, training, and professional guidelines were highlighted by healthcare providers. They generally had inadequate knowledge of the various types of substitution treatments, local services and support schemes that existed. Several emphasized that nurses and doctors without sufficient experience with the patient population lacked the prerequisites to understand the mechanisms of the treatment [48, 58, 59, 63]. Among healthcare providers, few had learned about, or acquired skills about, substance use disorders in their formal education, unless they had actively sought this type of knowledge themselves. They saw a need to incorporate this type of treatment into the education or specialization of health personnel through day courses, or conferences and continuing education as professionals [56, 59, 62]. They also emphasized the need for interdisciplinary competence and collaboration across systems as patients also needed support in areas outside health service’s domain [56, 61–63].

Lack of knowledge may have contributed to negative attitudes towards the patient group and OMT among healthcare providers. Several healthcare providers described OMT patients as problematic and difficult patients [61]. “Diversion of OMT drugs” and the use of other drugs in addition to treatment were also seen as a challenge [61, 65, 67]. Healthcare providers felt that offering OMT in their clinic could threaten the safety of other patients and employees [56, 61–63]. Several shared personal experiences and examples of how this uncertainty had influenced their professional decisions, and it was especially newly qualified doctors who wanted to avoid participating in the OMT system [58, 62, 63].

### Finding 4 Quality of communication between personnel and patients impacts patients’ engagement with treatment and treatment outcomes

The quality of communication and of relationships between patients and healthcare providers affects the quality of treatment and treatment outcomes. Patients and healthcare providers perceived that communication and patient-provider relationships were crucial for the quality of the OMT and as facilitators or barriers for treatment retention and outcomes. Healthcare providers and patients both reported that good communication and relationships could enable compliance, while poor communication and non-existent therapeutic relationships could negatively affect experiences of OMT for both patients and providers, and hinder treatment compliance.

Patients and healthcare providers believed that therapeutic relationships and good communication contributed to more openness about challenges, more targeted treatment, and better treatment compliance [43, 45–49, 51, 53, 54, 57, 65]. Patients described how it was important to be met with respect by providers, and that there was room for cooperation and openness about the treatment and problems that arose during the treatment [45–47, 49, 51, 53].

Patients wanted to be seen as a person instead of being identified with their condition [45, 64]. They wanted to actively participate in developing treatment plans and wanted their views on medication and dose to be considered [45–49, 54]. They emphasized that it was important to be able to be open about difficulties in treatment or relapse with therapists without this resulting in “punishment” [43, 44, 47].

Being prepared for and informed about what they could anticipate regarding the treatment was also highlighted as positive to be able to cope with challenges encountered during treatment [43]. Organizational and structural conditions of treatment also affected the interaction between patients and healthcare providers. The patients’ encounters with therapists also represented organizational factors, i.e. parts of the treatment process itself, as they took place and were shaped by certain settings and treatment structures (47, 49). Outpatient settings were seen by many as a safer and less stigmatized arena for meeting therapists than methadone centers and pharmacies [47, 64]. Here, many patients experienced being treated as “normal people” who received treatment and not “just” as one of the “drug addicts” (44, p 521). This setting gave more room to build relationship and trust with the therapists [47].

Many patients described that they felt that the provider-patient relationship was characterized by an asymmetric power distribution. The providers had power over the patients` lives and some patients were afraid of how this power could be used [45, 47, 54]. The sanctions patients faced if they did not report their use on the side or did not deliver urine samples on time made patients feel trapped and were perceived as incapacitating [45]. They described that there was little room for error in the system and that the control systems and fear of sanctions contributed to a lack of transparency with providers about problems and that relapses and problems were kept hidden [43–45, 47, 54]. This meant that some of the patients disclosed as little as possible to providers due to fear that this information could be used against them [45, 54]. They felt that providers focused on the negative rather than on what went well [45, 54]. This meant that patients adapted information that was provided to satisfy providers instead of being open about challenges, which in turn contributed to insufficient help to cope with the actual problems [45]. Several described that they did not feel heard by providers, and that they had no influence on treatment decisions [45, 54]. These negative encounters with providers affected the patients’ perceptions of the health services, influenced the treatment, and could contribute to the patients not being able to complete the treatment.

Communication and relationships between patients and providers were also topics among providers. Healthcare providers pointed out that it was important to build relationships with their patients to make room for trust and openness in the treatment situation [57, 63, 66]. Healthcare providers often found it challenging to discuss drug use with patients due to patients past experiences involving conflicts and fractured relationships with health and social services. This led to a lack of trust in both the providers and the overall system. This made the relational work challenging [57]. Stigmatizing attitudes among other healthcare providers (findings 1 and 2) were also highlighted by healthcare providers as a barrier to relational work [58, 59, 63, 64]. Providers’ unconscious negative attitudes, and in some cases fear, could affect the interaction with the patients negatively [63]. Some pointed out that the systems were not adapted for such relational work [57, 63]. Healthcare providers also suggested that they found office-based settings a better arena for relational work than methadone centers and pharmacies, as this setting was more adapted for individual follow-up [59, 64].

### Finding 5 Patients wanted help with many aspects of their lives not just medication

A consistent theme among patients was that OMT was much more than medication, and the outcomes they expected were just as often social as health-related. Patients portrayed their problems with substances as multifactorial including social, psychological and physical factors [43, 44, 46, 51, 65] and many described the need for a more holistic and multidisciplinary approach to treatment [43, 44, 48, 49, 51, 53]. In addition to opioid use, they wanted to address underlying problems that had contributed to their OUD problems [43, 44, 51]. They were concerned medication alone did not address the reasons why one began to use substances in the first place, but simply put a lid on these problems and feelings [44, 46, 51].

Many of the patients saw the treatment as an opportunity to bring about a change in their lives [45, 49, 51]. The patients’ experiences of their own need for OMT was influenced by what healthcare providers identified as “inner motivation for change” [43], which can also be understood as anything that patients thought OMT could help them achieve, such as to finish their education, obtain a driver’s license, being able to care for their families, or to improve their physical health [44, 46, 50, 65]. Patients described that they felt it was important to have a job or other interests to fill the “void” after cessation of substance use [44]. The fact that the treatment was adapted to individual needs was important to be able function in a job [45].

Being able to function in a job was identified by patients as leading to better self-esteem and better quality of life, as well as experiences of belonging, meaning and less stigma [43, 54, 64, 65]. It was important for them to be seen as contributors in society [44, 54, 64]. Having a job and responsibility for something contributed to a stability in life that in turn was expected to lessen the risk of relapse [44].

Many described how OMT helped them to resume a social life and improve relations with their family and friends [44, 45, 65]. Feeling like a “normal person” (in patients’ own words) ( [64] p, 521) was highlighted as very important for many of the participants, to which OMT in many cases contributed to [43, 54, 64, 65].

At the same time, several described that they felt tied to the treatment regime, which could contribute to poorer compliance [43–46, 48, 64–66]. This was particularly highlighted as a negative aspect of methadone treatment [43, 44, 52, 64]. Some wanted more freedom, as treatment could be an obstacle in working life, but also hampered opportunities to travel and have a “normal life” [45, 64].

Patients wanted a life away from the drug environment. However, they did not want to be isolated. In some OMT centres such as methadone centres, interactions with people from the drug community were almost guaranteed. Continued interactions with other patients who were still actively using drugs contributed to instability and this was a barrier to compliance to treatment [43, 44, 47, 64]. At the same time, they felt a need for human connection and loneliness was an issue. Not being able to socialize with old friends could entail social isolation and loneliness [51]. These kinds of social structures - which many expected to improve through OMT- affected the patient’s ability to carry out treatment. However, patients experienced that this was not considered in their treatment.

### Finding 6 Balancing positive expectations of OMT with treatment stigma

Patients continuously balanced positive expectations of OMT, and negative outcomes, (such as experiencing stigma from society and health care providers and feeling trapped in the treatment regime) especially those outcomes that were related to the stigma connected to OMT, their expectations of OMT and positive outcomes they hoped to achieve [44, 45, 51, 53, 56, 59, 64, 65]. Some patients found that the treatment had many positive non-health-related outcomes, while others found that these were lacking. However, patients generally expected such outcomes; These potential positive outcomes were a counterweight against the stigma they experienced in the system. For them to remain in OMT (with was potentially a negative experience), the positive outcomes had to outweigh the negative. They negotiated and reassessed this balance continuously. If the load of the stigma, and other negative outcomes exceeded the positive outcomes it became less profitable to follow the treatment regime. Freedom from the need to obtain drugs was weighted against the lack of freedom from being tied to the treatment regime. Improved quality of life was weighted against low satisfaction with the present life situation. Powerlessness was weighted against survival.

A “normal life” was a frequently used phrase (in patients’ own words (64, p 518)) that can be understood as the patients compromise between the stigma that comes with OMT [44, 64], and the positive outcomes they hoped to achieve in OMT. Some patients reported that the positive consequences of OMT contemplated the negative, often in the form of “normalization” [44, 53, 65]. Feeling “normal” represented a de-stigmatization from the stigma associated with OMT and substance abuse [64]. With this understanding, the reasons for whether the patient find OMT acceptable or not are complex, and dependent of various internal and external factors.

## Discussion

This QES of patients’ and health care providers’ experience of OMT synthesized findings from 24 primary studies published between 2010 and 2022. Our findings suggest that stigma, both external to the treatment setting and generated within OMT, posed significant barriers to individuals seeking and maintaining treatment. This stigma influenced health beliefs, creating a dilemma for individuals torn between avoiding societal judgment by concealing their addiction and accepting the stigma associated with seeking OMT. The inadequacy of knowledge and expertise among healthcare providers further hindered OMT access, treatment quality, and patient experiences, contributing to negative attitudes and stigma. Effective communication and positive patient-provider relationships were identified as crucial for OMT quality, influencing treatment compliance and outcomes. Patients’ expectations extended beyond health-related outcomes, emphasizing the need for a holistic, multidisciplinary approach. Despite positive OMT outcomes, patients continuously balanced these against negative experiences and stigma, highlighting the importance of achieving a “normal life” as a compromise between treatment benefits and associated societal judgments.

By using Andersen’s model as a framework, we identified several dynamic factors that facilitate or inhibit provision of OMT and patients’ access to and usage of OMT. These factors encompassed both individual and social aspects, incorporating various elements of the treatment process, such as stigma, communication with healthcare providers, provider competence, as well as patients’ own expectations and attitudes towards OMT factors.

We discovered that stigma cut across all four factors of the Andersen model. In this review we lean on Link and Phelans definition of stigma as the observable convergence of five dimensions experienced by a group: (1) Labelling as addicts or junkies, (2) Negative stereotypes (such as inaccurate beliefs held by healthcare providers), (3) Othering (the separation of Substance Use Disorder (SUD) from medical care), (4) Disparities in health and social outcomes, and (5) Limited access to economic and political power [68].

In our findings, stigma emerged as a main barrier, which can be understood as attitudes to OMT in the social context surrounding the individual. A recent systematic review that explored barriers to accessing opioid substitution treatment from the client perspective also identified social stigma as a major barrier. This included stigma from family and friends and from healthcare providers. Stigma was also related to lack of job opportunities, being a state-registered “addict” (p.9) and fear of being recognized at the treatment centre [24].

Stigma toward OMT, OMT patients and people who use drugs in general seem to both directly and indirectly contribute to OMT underutilization. Stigma acts as a contextual and individual barrier at the forefront of treatment that increase the threshold for patients to start in OMT. Negative attitudes in society increase the threshold for patients to start in OMT, both because they want to avoid other people’s prejudices, but also because many themselves have internalized this stigma.

Stigma from OMT providers themselves was a barrier to forming a good therapeutic relationship with patients, which in turn increased the risk of patients discontinuing treatment. The stigma was reproduced by the OMT system, which allows it to be counted as an outcome beyond human relationships between the patient and healthcare providers. Lack of knowledge can lead to stigma and reinforcement of stereotypes. Shidlansik and colleagues found a correlation between the presence of stigma, and the extent of education and knowledge about methadone maintenance treatment, among providers in OMT clinics and the social services in Israel [69]. This indicates that more educational interventions, among personnel, may benefit people who use opioids and improve the overall quality of treatment for OUD.

Allen and colleagues argue that to effectively treat OUD and address stigma in the healthcare system, it is a perquisite that clinicians are given comprehensive education about OUD as a chronic disease and that evidence-based treatment of OUD is incorporated into medical curricula. Furthermore, they emphasize that it is important to give better access to effective treatment, raise awareness about the condition and develop a workforce that understands the patients and their problems and that are able to meet them with compassion instead of distrust [70].

The studies we included only indirectly focused on the organization of the services, however, both patients and healthcare providers point to contextual factors that influence their experiences of OMT, such as organization of services, knowledge and expertise among healthcare providers and access to other health services. The ways that OMT were physically and organizationally facilitated may have a considerable impact on how patients experience OMT and can lead to patients feeling more stigmatized, sometimes to an even greater extent than when they were outside OMT, as described in relation to standing in line in methadone centers and pharmacies. The way OMT is organized can help produce and reproduce stigma and contribute to negative attitudes in the society. Organizational and structural conditions also affected the interaction between patients and healthcare providers. According to our results it seems that delivering methadone and buprenorphine through less stigmatizing and less burdensome outlets (e.g., standing in line at a specialized clinic versus a pharmacy) could help change the cultural narrative around OMT and OMT patients and reduce stigma.

Mehta and colleagues published a systematic review om the effect of interventions to reduce mental illness-based stigma and discrimination. They found modest evidence for the effect of interventions to increase knowledge and reduce stigmatizing attitudes with more than four weeks follow-up. However, to our knowledge there is few research studies exploring stigma-reducing interventions targeting opioid dependence outside and within the OMT system. Researchers have suggested anti stigma interventions such as public awareness campaigns, education of healthcare providers, family therapy for affected families, and use of community meetings [55, 71].

### Strengths and limitations

Our systematic approach is a major strength of this review. We have used a systematic method of searching, identifying, and analyzing relevant studies. We have assessed our certainty in the findings and presented transparent assessments of how much confidence decision-makers can place in each finding in this review. Nevertheless, a limitation to this review is the possibility that we may have excluded studies that could have given other findings, perspectives, and substantial knowledge. There are also large variations in structure and content of the OMT programs between the countries in which the included studies are conducted. The programs differ in use of primarily medications, in type of follow-up and in resources used on psychosocial rehabilitation and treatment of comorbid disorders. Notably, we had no findings that related to stigma, discrimination, or prejudice that were not directly substance- or OMT-related. Yet a large body of evidence exists that has identified race and ethnicity [72], gender and sexual minority status [73], disability, age [74] and increasingly, neurodiversity status [75], as relevant to treatment outcomes in mental health and other types of health treatment, due to discrimination within the healthcare system and to the vulnerabilities incurred beforehand because of this minority status. It is reasonable to expect that various sources and types of stigmas may interact when people are in need of or receive OMT. An intersectional approach to stigma would be quite valuable, as Turan et al. have called for [76]. The composition of this research team was majority non-racialized, majority non-LGBTQ, and all highly educated and neurotypical, and it might be possible that a more heterogenous group of reviewers might have picked up more subtle layers of discrimination and shame in the primary research. We believe it more likely that the primary research itself was not attentive to intersectionality. Intersectional stigma should be a prioritized research topic going forward because treatment and guidelines must have the evidence base to address vulnerabilities effectively and inter-sectionally.

## Conclusion

Understanding contextual and individual factors, health behaviours and outcomes in OMT that influence initiation is important, as opioid use disorder is a serious medical and social problem. The findings of this QES indicate that treatment programs may be more beneficial to the clients if they focused on reducing stigma in society and among OMT providers. Treatment programs should also focus on what works for patients: bridging the gap between treatment needs and treatment provision, reducing barriers to treatment uptake, and increasing other patient-identified facilitators. Parallel initiatives should focus on increasing treatment knowledge among healthcare providers, client`s perspective utilization and understand the context of their family members and offer a more holistic and flexible treatment setting.

### Electronic supplementary material

Below is the link to the electronic supplementary material.

Appendix 1

Appendix 2

## Data Availability

The datasets generated and/or analyzed during the current study are available from: https://isoq.epistemonikos.org/preview/isoq/d9e514469c0ed9cd/600ade199316674e11b1e4aa/public.
