# Supplementary material for: Patient’s and healthcare provider’s experiences with Opioid Maintenance Treatment (OMT): a qualitative evidence synthesis
Source: BMC Health Serv Res. 2024 Mar 13;24:333. doi: 10.1186/s12913-024-10778-7 (PMC10938774; doi:10.1186/s12913-024-10778-7)
Supplement: Supplementary file 2 — Appendix 2 [file 12913_2024_10778_MOESM2_ESM.docx]

**Appendix 2: Description of included studies**

| **Study, year (country)** | **Participants** | **Setting** | **Methods** |
| --- | --- | --- | --- |
| Bates & Martin-Misener 2021, (Canada) | 18 participants:  nurse practitioners (n= 5), physicians (n=5), and stakeholders including members of professional regulatory bodies and government, academics, and other clinicians (n=8) | Primary care, family and specialist practices, professional regulatory bodies, health organizations and government, universities, and other clinics | Phone semi-structured interviews, thematic analysis |
| Belseth 2016 (Norway) | 14 milieu therapists | One OMT clinic | Focus group interviews Hermeneutic-phenomenological approach. |
| Bishop 2019 (New Zealand) | 7 OMT patients, 5 men and 2 women aged 25 - 65 | Buprenorphine/naloxone treatment | Qualitative interviews, thematic analysis |
| De Maeyer 2011 (Belgium) | 25 OMT patients aged 26 – 46 years | Methadone maintenance treatment | Qualitative interviews, thematic analysis |
| Gordon 2011 (USA) | 61 providers | Veterans’ Health Administration (VHA) facilities | Semi structured telephone interviews |
| Granerud 2015 (Norway) | 7 OMT patients’, 3 men and 4 women, aged 26 - 49 years, with an average of 41 years. | OMT clinics | Qualitative interviews, grounded theory-inspired approach |
| Green 2014 (USA) | 101 clinicians and clinicians–administrators | Primary and specialty care | Qualitative interviews, analyses informed by existing models of adoption of new pharmacotherapies and classical diffusion theory. |
| Harris 2015 (USA) | 13 OMT patients, 5 doctors, 3 nurses, 2 medical directors, 2 psychologists, 2 program directors, 1 counselor, 1 therapist, and 1 pharmacist | OMT clinics | Observation and qualitative interviews, discourse analysis |
| Hewell 2017 (USA) | 11 persons with an opioid use disorder, 64 % women | OMT clinics | Focus group interviews, grounded theory |
| Johnson 2014  (Sweden) | 25 professionals | Eight OMT clinics | Qualitative interviews, qualitative textual analysis |
| Korthuis 2010 (USA) | 29 OMT patients with HIV infection and opioid dependence | Buprenorphine maintenance therapy | Qualitative interviews, thematic analysis |
| Lachapelle 2021  (Canada) | 27 people with former or current opioid use disorder | Treatment  facilities and community organizations. | Focus group interviews thematic analysis, |
| Livingston 2018, (Canada) | 20 primary care physicians | Primary care settings | Qualitative interviews, interpretive approach |
| Marchand 2020,  (Canada) | 30 OMT patients | One OMT clinic | in-depth interviews, constructivist grounded theory approach |
| Notley 2014  (UK) | 29 OMT patients and 55 professionals | Four OMT clinics | Semi-structured interviews, grounded theory approach |
| Notley 2015  (UK) | 27 OMT patients, 9 women and 18 men (mean age 47 years) and 10 professionals | One OMT clinic | Individual interviews, grounded theory approach |
| Richert 2015 (Sweden) | 27 (7 women and 20 men aged 24-53 years) opioid users who have treated themselves with methadone or buprenorphine for at least three months | OMT clinics and needle exchange sites | In-depth interviews, qualitative textual analysis. |
| Silva & Andersson 2021  (Sweden) | 19 OMT patients (16 men and three women) | One OMT clinic | One-on-one interviews grounded theory approach, |
| Sohler 2013 (USA) | 38 opioid-dependent persons (5 women and 33 men with average age of 44 years) | 3 needle exchange  programs | Focus group interviews |
| Tanner 2011 (UK) | 12 OMT patients with experience from both Methadone and buprenorphine treatment | OMT clinics | Structured interviews and written narrative accounts |
| Toft 2013  (Norway) | 7 OMT patients (4 women and 3 men aged 26-49 years) | OMT clinics | Semi-structured individual interviews (face-to face and by Skype), grounded theory approach |
| Van Hout et al. 2018  (UK) | 11 experts and key stakeholders involved in OAT (5 women and 6 men) | statutory and non-statutory agencies involved in OMT | A focus-group interview, content analysis |
| Woo et al. 2017  (Canada) | 18 OMT patients (12 women and 6 men with mean age of 36 years) | Two methadone clinics | Semi-structured individual interviews, Thematic content analysis |
| Yadav et al. 2019  (UK) | 24 pharmacists (14 women and 10 men) | Pharmacies | Semi-structured individual interviews, interpretative phenomenology analysis |
| OMT: Opioid Maintenance Treatment | | | |
